# Prolonging the Durability of Maritime Constructions through a Sustainable and Salt-Resistant Cement Composite

**DOI:** 10.3390/ma16216876

**Published:** 2023-10-26

**Authors:** Mohamed Heikal, Mohamed A. Ali, Djamel Ghernaout, Noureddine Elboughdiri, Badia Ghernaout, Hazem I. Bendary

**Affiliations:** 1 Chemistry Department, Faculty of Science, Benha University, Benha 13518, Egypt; mohamed.heikal@fsc.bu.edu.eg; 2 School of Biotechnology, Badr University in Cairo (BUC), Badr City 11829, Egypt; mohamed.ahmed_ali@buc.edu.eg; 3 Chemical Engineering Department, College of Engineering, University of Ha’il, P.O. Box 2440, Ha’il 81441, Saudi Arabia; djamel_andalus@yahoo.fr; 4 Chemical Engineering Department, Faculty of Engineering, University of Blida, Blida 09000, Algeria; 5 Chemical Engineering Process Department, National School of Engineers Gabes, University of Gabes, Gabes 6029, Tunisia; 6 Mechanical Engineering Department, Amar Tlidji University of Laghouat, Laghouat 03000, Algeria; badiagh@gmail.com; 7 The Higher Institute of Engineering, Chemical Engineering Department, El-Shorouk Academy, Shorouk City 11837, Egypt; hazemibrahem31@yahoo.com

**Keywords:** seawater, sulfate attack, blended cement, compressive strength, chloride attack, combined water content CWC

## Abstract

This research investigates the long-term resilience of an environmentally friendly cement blend comprising Egyptian Ordinary Portland Cement OPC and Ground-Granulated Blast Furnace Slag GGBFS when exposed to a corrosive seawater environment. This scientific investigation explores the effects of exposure to seawater on various properties of cement pastes, encompassing parameters such as free lime content (FLC), chemically combined water content (CWC), bulk density (BD), total porosity (ϕ), total sulfate content, total chloride content, and compressive strength (CS). By contrast, Differential Thermal Analysis (DTA), FT-IR spectroscopy, and X-ray diffraction (XRD) analysis can be utilized to investigate the influence of exposure to seawater on the hydration products of GGBFS cement pastes over a period of up to one year. This analytical approach offers valuable insights into the alterations that occur in hydration products and their resilience when subjected to seawater conditions. The results obtained from this investigation reveal that all cement pastes incorporating GGBFS exhibit heightened resistance to deterioration in seawater, with slag cement containing 60 wt. % GGBFS and achieving a notable compressive strength of 85.7 Mpa after one year of immersion in seawater. These findings underscore the capacity of these cement blends to effectively withstand challenges in durability in marine environments.

## 1. Introduction

Researchers have extensively studied the multifaceted process of deterioration in concrete structures caused by exposure to seawater, as it can lead to significant economic and environmental consequences [1,2,3,4,5,6].

External sulfate attacks can cause concrete damage through two different mechanisms. The first is chemical, where sulfate ions react with concrete’s cementitious components, leading to an expansion driven by ettringite formation and/or gypsum. The second is physical, where the development and internal stress caused by the chemical reactions result in concrete cracking. Consequently, concrete becomes more permeable, making it easier for aggressive compounds to penetrate. Additionally, research conducted by different scholars has shown that the adsorption of sulfate ions in a free state onto the particles of calcium-silicate-hydrate can also contribute to the physical attack mechanism [7,8,9,10,11,12].

The phenomenon of an external sulfate attack has been extensively studied and documented in the literature, even if there are still unanswered questions and ongoing concerns, especially regarding the mechanisms underlying such a phenomenon. The sulfate attack complexity can be primarily attributed to the wide variety of ions, including calcium, sodium, and magnesium sulfate, which can potentially affect the composition of the paste [13]. The role of gypsum in causing significant expansion and damage to cement paste when exposed to sodium sulfate is a topic that is still being debated and is not yet fully understood. The exact pathways by which gypsum contributes to these effects are uncertain [7,8,13,14]. Investigators have suggested that (i) the formation of gypsum during the sodium sulfate attack could be responsible for surface deterioration and a decrease in mechanical strength, and (ii) the presence of gypsum plays a significant role in adverse effects observed during the sodium sulfate attack, leading to the degradation of surfaces and a reduction in the overall strength of the affected material [15]. Gypsum could fill the air pockets in cement paste, causing the cement to expand in size [16,17,18,19,20]. Considering that both Ca(OH)_2_ and 4CaO·Al_2_O_3_·13H_2_O are significant products of the sulfate reaction involving C3S, C2S, and C3A during cement hydration, any strategy aimed at inhibiting the formation of both Ca(OH)_2_ and 4CaO·Al_2_O_3_·13H_2_O has the potential to enhance the resistance of concrete against sulfate attack. Various methodologies have been investigated to improve the resistance of concrete to water penetration, including using clay brick powder as an additive [21], regulating the water-to-cement ratio [22], substitution with fly ash [23,24,25], blast furnace slag [26], and utilizing silica fume [23,27]. These materials interact with Ca(OH)_2_, creating more C-S-H gel and reducing the amount of gypsum produced during the sulfate reaction. This takes place because such materials contain high silicates and absorb portlandite [28].

Many nations and organizations desire to achieve carbon neutrality by 2050. This is in response to the urgent need to address the effects of the phenomenon of global warming and alterations to the climate [29]. Carbon capture and storage technologies hold promise as practical approaches to address carbon emissions [30]. Cementitious materials have significant potential for securely and permanently capturing and storing CO_2_, particularly in construction applications [31,32,33,34]. ASTM C595 [35] defines a pozzolan as a material containing siliceous or aluminous components. These components, on their own, lack significant cementitious properties. However, when exposed to moisture at ordinary temperatures, they undergo a chemical reaction with calcium hydroxide, resulting in the formation of compounds possessing cementitious characteristics. Numerous established pozzolanic materials have been derived from industrial and agricultural byproducts, for instance, materials like silica fume, fly ash, and rice husk ash. Additionally, materials like GGBFS, which is characterized by a significant glassy phase content and activated clays, can be employed as a substitute for cement [36]. The production of Portland cement requires significant energy, prompting efforts to investigate alternative materials that can replace cement. Fine pozzolanic materials have been identified as suitable substitutes for cement in concrete, as they can improve bulk density (BD) by filling voids and contribute to strength development, making them a promising choice for reducing cement usage in concrete production [37]. GGBFS, an industrial by-product in Egypt, is a glassy material from rapidly cooling molten iron during steel production. It contains a substantial amount of amorphous calcium, silica, and alumina that is ideal for construction [38]. In total, 65% of high-abundance slag (530 million tons/year) was used as a construction sector cementitious material [39]. Using GGBFS in construction enhances durability, prevents early thermal cracking, and is cost-effective [40].

This study assesses the performance of cement paste made with GGBFS-rich cement from Egypt when exposed to harsh water conditions. Multiple blended cement formulations were developed through the partial substitution of OPC with an equivalent quantity of GBFS. To assess their durability, an examination of chemical factors such as CWC, FLC, total chloride, and total sulfate was conducted. An evaluation of physicomechanical properties involves the monitoring of BD and compressive strength CS over varying immersion durations (1, 3, 6, 9, and 12 months). Furthermore, the effects of exposure to harsh environmental conditions on these materials were studied utilizing Differential Thermal Analysis (DTA), Fourier Transform-Infrared (FT-IR), and X-ray diffraction (XRD) methodologies.

## 2. Materials and Methods

### 2.1. Materials Characterization

In this study, the primary materials consisted of OPC obtained from the Alexandria Cement Company and GGBFS sourced from the Iron and Steel Company located in Helwan, Egypt. Two types of water were employed as follows: freshwater for mixing and curing the specimens until testing and Red Sea seawater from Ain Sukhna Beach (Suez Governorate, Egypt) to assess the resistance of blended cement specimens to saltwater. Seawater contains various ions, including Na^+^, Mg^2+^, Cl^−^, and SO_4_^2−^, along with tiny amounts of potassium ions (K^+^), calcium ions (Ca^2+^), bicarbonate ions (HCO^3−^), and bromide ions (Br^−^) [41]. The average salinity of seawater is approximately 3.5%, which is equivalent to 35 g of dissolved salt per liter [42].

**Figure 1:**
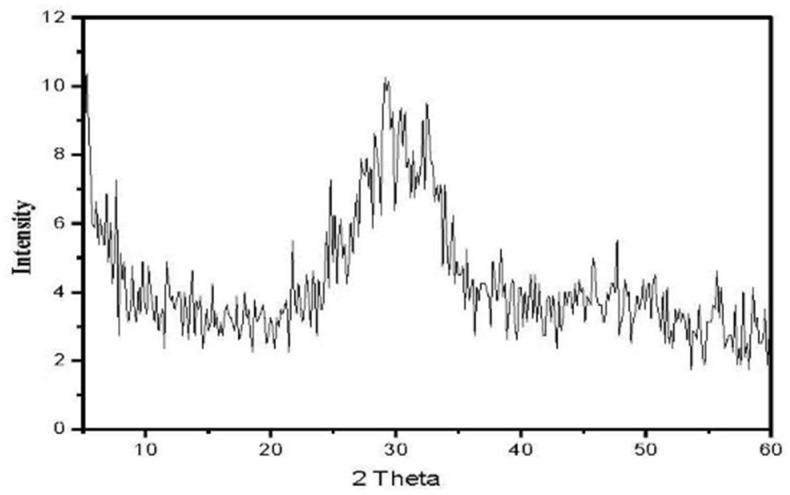
XRD pattern of GGBFS.

The materials were subjected to a 24 h drying process at 110 °C in an electric dryer to eliminate any residual moisture. The GGBFS underwent a fine-grinding process utilizing a laboratory steel ball mill with a capacity of 5 kg. The Blaine surface area of both OPC and GGBFS was assessed through the utilization of the ASTM technique [43]. The results showed a surface area of 3000 ± 50 and 4000 ± 50 cm^2^/g for OPC and GGBFS, respectively. The data presented in Table 1 provide the chemical analysis of OPC and GGBFS. The XRD technique has been used to confirm the amorphous structure of GGBFS in previous work [44], as depicted in Figure 1. Exhibiting a peak in the diffraction pattern within the 30 to 40-degree range, this observation signified the existence of amorphous glassy phases.

### 2.2. Specimen Preparation

The specific composition of GGBFS cement is accessible in Table 2. The components of each blend were mixed in a porcelain ball mill for one hour, and complete homogeneity was achieved using a mechanical roller mill. The hydration procedure for the paste samples was conducted by adhering to ASTM guidelines [45], employing the prescribed water-to-cement ratio, which is expressed as a percentage of the standard consistency (W/C, %), as outlined in Table 2. Freshly prepared pastes were shaped into cubes with dimensions of one inch. These specimens were subjected to 100% relative humidity at a controlled temperature of 23 ± 2 °C for a duration of 24 h, after which they were demolded subsequently. The specimens were immersed in tap water and subjected to a curing period of 28 days, which was considered as the initial point (zero time). Then, they were immersed in seawater for the duration of the testing period (1, 3, 6, 9, and 12 months) [46,47].

### 2.3. Test Procedure

Before conducting the CS tests, BD and ϕ measurements were carried out. The specimens were weighed both in a suspended state within the water and in the air precisely when they attained a condition known as saturated surface dryness. These measurements were carried out on a minimum of three identical cubes sharing the same blend composition and curing duration. The density was computed using the equations outlined in the documented references [48]. CS testing was conducted in conformity with ASTM specifications [49].

To stop the hydration of cement pastes, after compressive strength testing, 10 g of the representative samples were ground and placed in a beaker with a 1:1 methanol–acetone mixture. This mixture was mechanically stirred for one hour; then, it was filtered, and the solid was washed multiple times with the stopping solution and diethyl ether. The resultant solid material was subjected to a one-hour drying period at 70 °C, subsequently gathered, placed into polyethylene bags, sealed, and stored within desiccators for further analysis [50,51].

The (CWC, %) in each blend was ascertained by calculating the percentage of weight loss upon ignition (LOI) with respect to the weight of the samples and following the ignition process. The calculation of CWC was adjusted to account for the water originating from free portlandite present in each sample, as detailed in references [52,53]. The (FLC %) was measured using the ammonium acetate method [54].

The sulfate and chloride content were evaluated using established protocols [46,47]. The powder XRD method was employed. A Philips diffractometer PW 1730 (Philips Electronic Instruments Co., Model PW 1730, Eindoven, The Netherlands) was used, utilizing Cu kα radiation with a wavelength (λ) of 1.5418 Å. The scan step size for 2θ was employed, and the collection time for each data point was 1 s. The scanning range for 2θ was set from 5 to 55 degrees.

The X-ray tube operated at a 40.0 KV voltage and 40.0 mA current. To identify the crystalline phases in a sample based on its XRD pattern, the JCPDS database was consulted online [55]. DTA was conducted under atmospheric conditions using a DT-30 Thermal Analyzer manufactured by Shimadzu Co., based in Kyoto (Japan). In each experiment, approximately 50 mg (with a particle size of 76 μm) of calcined alumina was used as an inert material. The finely pulverized hydrated cement paste was meticulously placed into a crucible made of platinum–rhodium at a consistent heating rate of 20 °C per minute, which was applied uniformly during the experimental procedure [56]. For FT-IR spectroscopic analysis, the specimens were fabricated by crafting potassium bromide (KBr) disks containing alkali halides and subsequently subjected to analysis employing a Genesis FT-IR spectrometer. The analysis covered the 400–4000 cm^−1^ range, and each sample underwent 256 scans at a resolution of 2 cm^−1^ [57].

## 3. Results and Discussion

### 3.1. Chemically—Combined Water Content (CWC)

**Figure 2:**
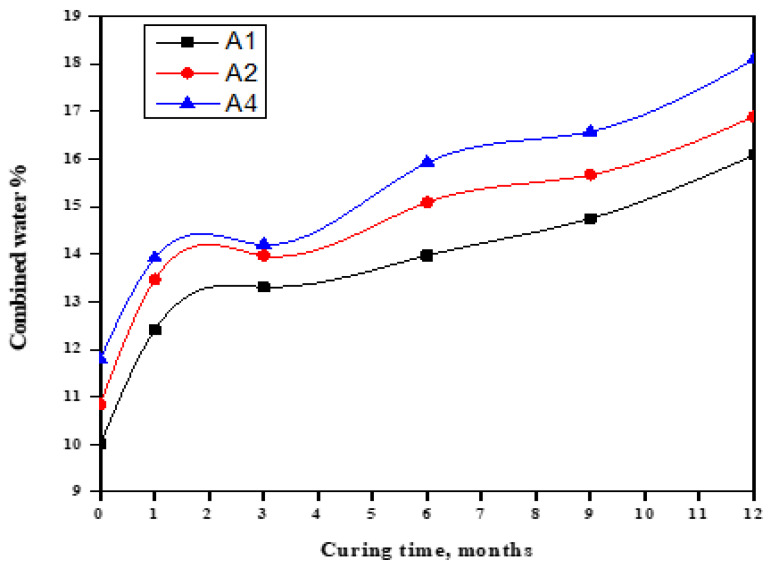
CWC of GGBFS rich blends in seawater up to one year.

Figure 2 illustrates the changes in the CWC of hardened GGBFS cement pastes during their immersion in seawater. It can be observed that the CWC increased as the immersion duration progressed: a trend observed across all cement pastes [58]. This phenomenon could be ascribed to the sustained hydration of the primary cement clinker phases and the slag component, particularly in the presence of MgSO_4_, MgCl_2_, and NaCl. These compounds accelerate the hydration process of cement phases, resulting in the formation of additional hydrated products with increased water content. As the proportion of imported GGBFS in the cement mixture was raised, the combined water content gradually diminished. This reduction was primarily due to a decrease in the C3A content of OPC, which plays a pivotal role in the formation of either calcium hydroxide (CH) or ettringite, both of which have a relatively high combined water content. After one month, there was a noticeable surge in the combined water content of all cement pastes, which is a result of the rapid activation of chloride (Cl^−^) and sulfate (SO_4_^−^) ions, which generate more hydration products. From one month up to one year, there was a slight incremental increase in the combined water content.

### 3.2. Free Lime Content (FLC)

**Figure 3:**
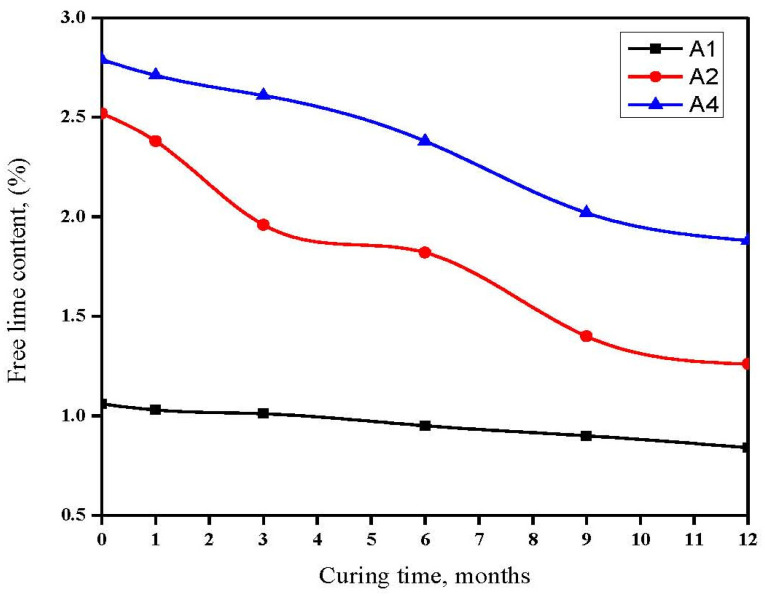
FLCs of GGBFS blend in seawater for up to one year.

The graph in Figure 3 depicts the FLC of various cement pastes during immersion in seawater for a duration of up to one year. The results illustrate a gradual decline in FLC for GGBFS cement pastes over the course of one year when submerged in seawater. This phenomenon can be attributed to the pozzolanic reaction initiated by GGBFS and catalyzed by lime, leading to the formation of C-S-H gel or C-A-H and C-A-S-H phases. As the quantity of GGBFS in the mixture increased, the FLC diminished due to the high pozzolanic characteristics of GGBFS [44].

Conversely, the FLC of cement pastes exposed to seawater for up to one year increased with a higher OPC content but decreased with a greater GGBFS content. This increase was primarily a result of the release of free lime during OPC hydration. Conversely, the pozzolanic reaction of GGBFS (A4) demands a lower FLC, contributing to an overall reduction in FLC when GGBFS is present.

MgSO_4_ can react with calcium hydroxide (Ca(OH)_2_) in the presence of seawater to form gypsum (CaSO_4_·2H_2_O) and magnesium hydroxide (Mg(OH)_2_), which can influence the chemical composition and durability of cementitious materials. This phenomenon elucidates the observed low FLCs in pastes submerged in fresh seawater. Additionally, MgCl_2_ reacts with a portion of Ca(OH)_2_ to form Mg(OH)_2_, further lowering the FLC.

### 3.3. Bulk Density (BD)

**Figure 4:**
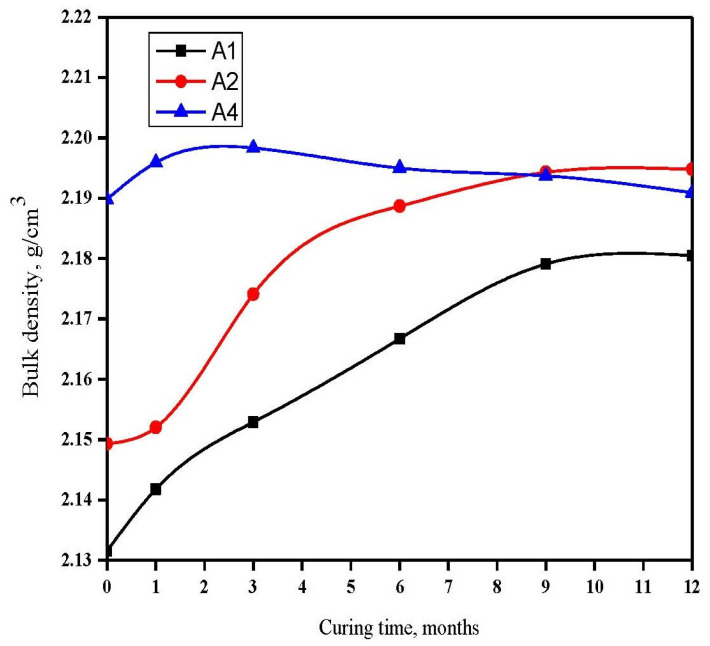
BD of GBFS rich blends immersed in seawater for up to one year.

Figure 4 illustrates the BD values of hardened GGBFS cement pastes immersed in seawater for up to one year. The results indicate that the BD of Mix A_4_ initially increased for up to three months and then gradually decreased for up to one year. The early-stage increase in BD was predominantly due to the activation of cement by chloride and sulfate ions in the seawater. This led to the formation of hydrated products that filled some of the open pores. As the immersion time extended beyond three months, the quantity of ettringite and chloroaluminate hydrate increased. The sharp rise in BD at early ages was primarily due to the faster reaction of OPC compared to GGBFS.

Ettringite possesses a substantial volume, resulting in a decrease in BD for up to one year. The initial rise in BD of Mix A4 is primarily due to a higher BD of OPC compared to GGBFS. The subsequent decrease in BD after three months up to one year is mainly due to a reduction in hydration products and total porosity, which facilitates the penetration of aggressive ions, leading to expansion or softening.

By contrast, an increase in GGBFS content enhances the chemical resistance of cement pastes. This can be attributed to the reaction between GGBFS and liberated lime, which results in the consumption of free lime and, consequently, no opportunity for lime to react with Cl^−^ and SO_4_^2−^. Thus, as the GGBFS content increases, the durability of the cement paste also increases [47].

C-S-H gel exhibits a high BD; therefore, an increase in the OPC content increases the C-S-H gel and BD. This is primarily because the hydrated phases of slag cement provide a lower BD than the hydration products of OPC pastes.

### 3.4. Total Porosity (ϕ)

**Figure 5:**
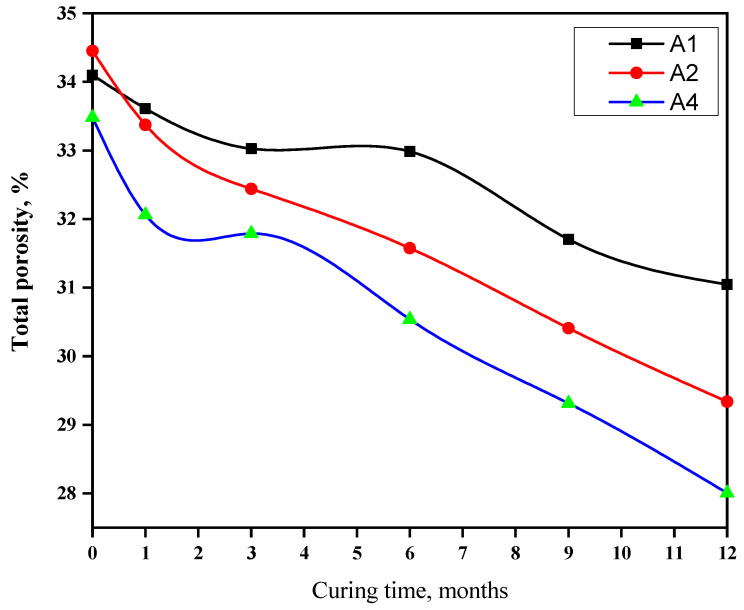
Total porosity of blends rich in GGBFS during immersion in seawater for a duration of up to one year.

Figure 5 illustrates the ϕ of the hardened cement pastes after immersion in seawater for durations of 1, 3, 6, 9, and 12 months. These findings revealed a gradual reduction in the (ϕ) of hardened cement pastes as the immersion time progressed. This could be attributed to the precipitation of hydrated products within the available pores of cement pastes. It was observed that an increase in the content of GGBFS could lead to a decrease in the interaction between liberated lime and seawater salt, resulting in the formation of magnesium silicate hydrate, chlorosulfoaluminate hydrate, gypsum, and other products that contribute to an increased ϕ.

### 3.5. Total Sulfate Content

**Figure 6:**
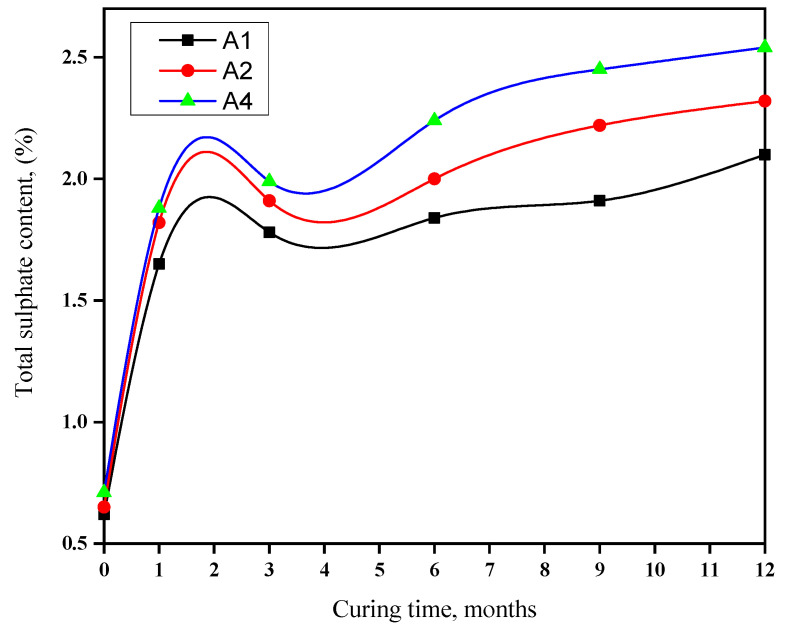
Total sulphate blends rich in GGBFS during immersion in seawater for a duration of up to one year.

Figure 6 presents the total sulfate concentrations in various cement pastes during their immersion in seawater for a period of up to one year. The increase in total sulfate levels primarily arose from the interaction of C3AH6 and Ca(OH)_2_ in cement pastes with SO_4_^2−^ ions in seawater, leading to the formation of AFt and AFm phases. Additionally, MgSO_4_, which is present in seawater, reacts with C-S-H gel and CH, resulting in the generation of gypsum, which subsequently reacts with CAH to produce sulfoaluminate hydrates such as AFt or AFm.

Observations reveal that the total sulfate content rises with increasing OPC content across all immersion durations in seawater. This trend is primarily attributable to higher concentrations of C3A and Ca(OH)_2_ in OPC, both of which are susceptible to sulfate ion reactions. The results from the total sulfate analysis suggest that cement pastes with higher proportions of GGBFS exhibit enhanced resistance to sulfate attack. As the quantity of slag in the mix increases, the paste’s resilience in aggressive environments improves. Consequently, replacing OPC with GGBFS or other pozzolanic materials may enhance resistance to sulfate attacks. These findings align with the observations from FLCs and compressive strength tests.

### 3.6. Total Chloride Content

**Figure 7:**
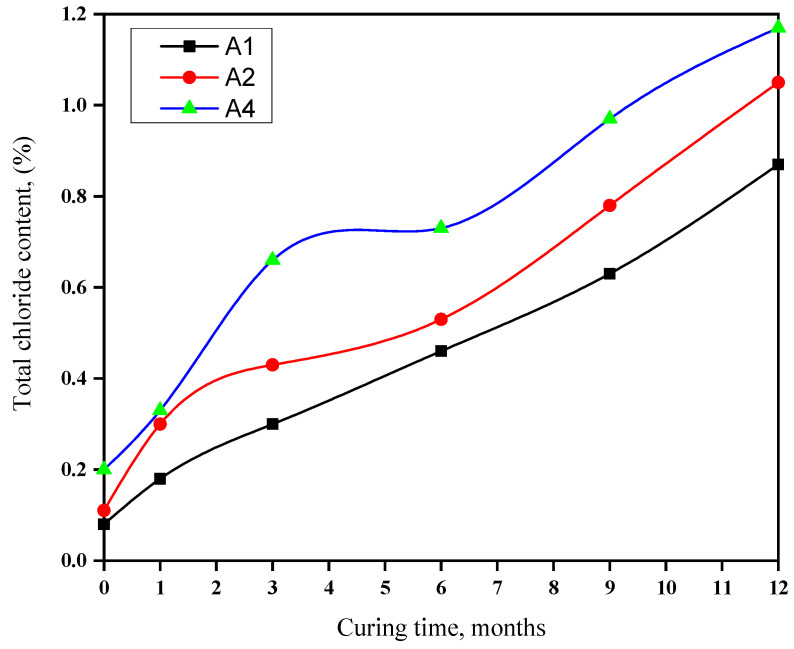
Total chloride of blends rich in GGBFS during immersion in seawater for a duration of up to one year.

Figure 7 shows the total chloride contents in cement pastes immersed in seawater for 1 to 12 months. Chloride content increases gradually due to Cl^−^ reacting with C3AH6 and CH, forming chloroaluminate hydrates. A sharp increase results from fast Cl^−^ penetration, forming chloroaluminate hydrates. The OPC content influences chloride diffusion; GGBFS enhances resistance to chloride attacks. GGBFS augments chloride binding due to alumina, hydrotalcite, reduced sulfate, and the C-A-S-H phase. Factors affecting chloride binding include slag content, the w/b ratio, composition, and curing temperature. Slag-rich cement improves sulfate resistance due to C-S-H gel formation, reduced permeability, sulfate ion diffusion, and ettringite stability. Seawater is more aggressive than chloride or sulfate solutions as sulfate and chloride ions form hydrates in cement pastes. Slag enhances chloride binding via alumina, hydrotalcite, sulfate reduction, and C-A-S-H phase formation [59,60,61,62,63,64,65].

### 3.7. Compressive Strength (CS)

**Figure 8:**
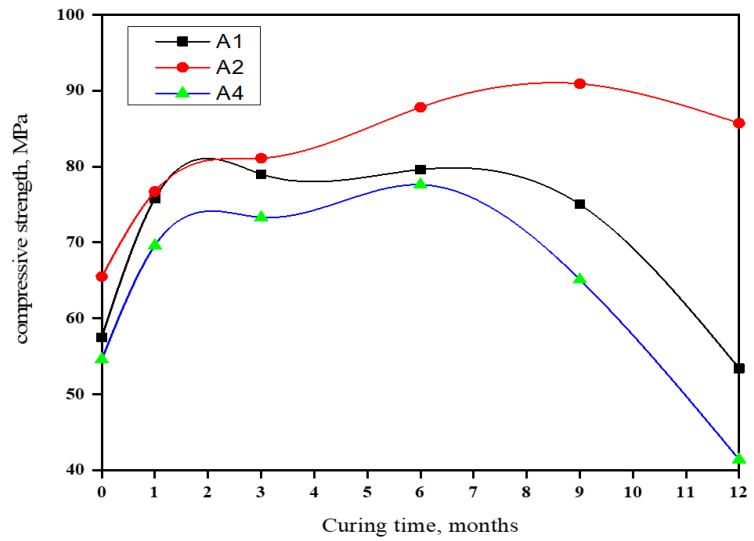
Compressive strength of blends rich in GGBFS during immersion in seawater for a duration of up to one year.

Figure 8 shows the (CS) of seawater-immersed slag-rich cement pastes for one year. A2’s strength increased for up to 9 months, then declined. A1 and A4’s strength rose for six months due to MgSO_4_, NaCl, and MgCl_2_ accelerating the cement pastes. Mg^2+^ ions enhanced C-S-H gel crystallization and cement strength. This led to C-S-H gel, chloroaluminate, and sulfoaluminate hydrate formation, with increased C-S-H gel in the first six months. A1 (70% GGBFS) had a high porosity, enabling Cl^−^ and SO_4_^2−^ migration, forming low-strength hydrates. A1 (30) and A4 (60) wt. % OPC weakened after six months due to more C3A and CH forming low-strength hydrates. A2 (60% GGBFS, 40% OPC) was the optimum pozzolanic cement, peaking at nine months. After one year, A1, A2, and A4 had compressive strengths of 53.4, 85.7, and 41.4 MPa, respectively. NaCl boosted Ca(OH)_2_ solubility, improving C-S-H gel stability and strength. Sulfate ions caused internal stresses, cracking, and AFt formation. MgSO_4_ reacted with the formed Ca(OH)_2_, forming gypsum, and Mg(OH)_2_, which has low solubility and precipitates as a gel. At later ages, it attacked C-S-H to form Ca(OH)_2_ and magnesium silicate hydrates, which have no binding properties, while MgCl_2_ attacked C3A and CH, softening the cement [66,67]. The 40:60 wt. % OPC/GGBFS blend exhibited higher compressive strength.

### 3.8. FT-IR Spectroscopy

**Figure 9:**
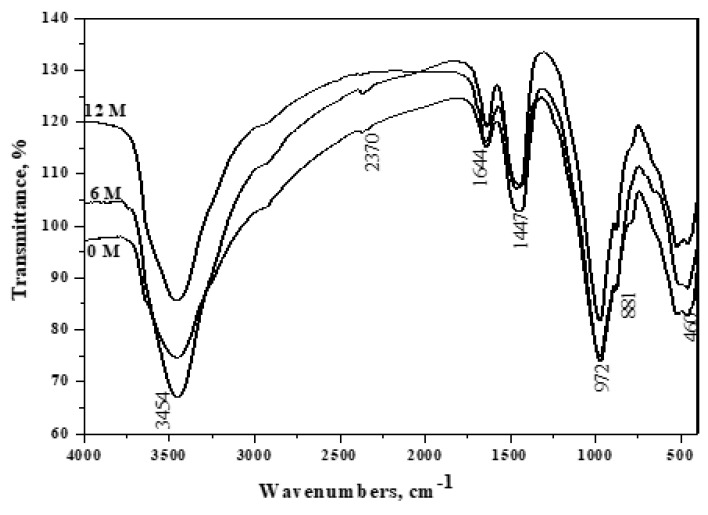
FT − IR spectroscopy of A1 (30% OPC + 70% GGBFS) immersed in seawater for 0, 6 and 12 months.

**Figure 10:**
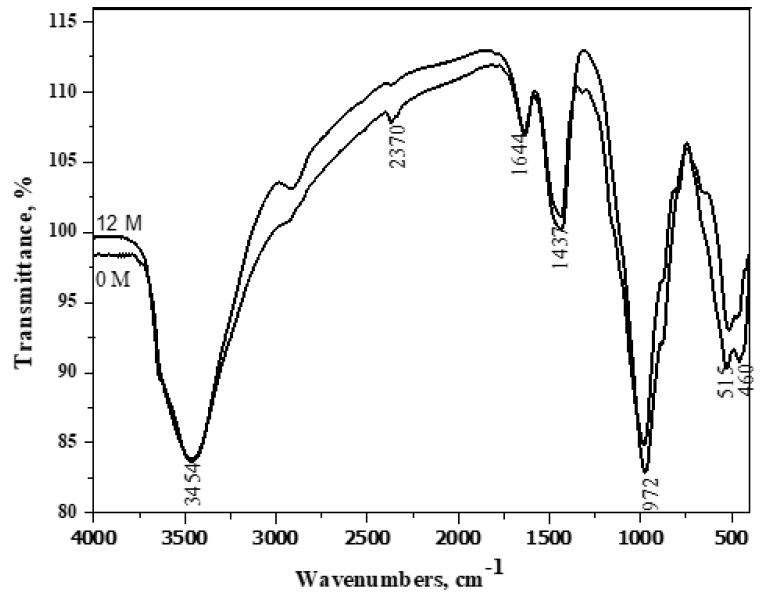
FT − IR spectroscopy of A2 (40% OPC + 60% GGBFS) immersed in seawater for 0 and 12 months.

**Figure 11:**
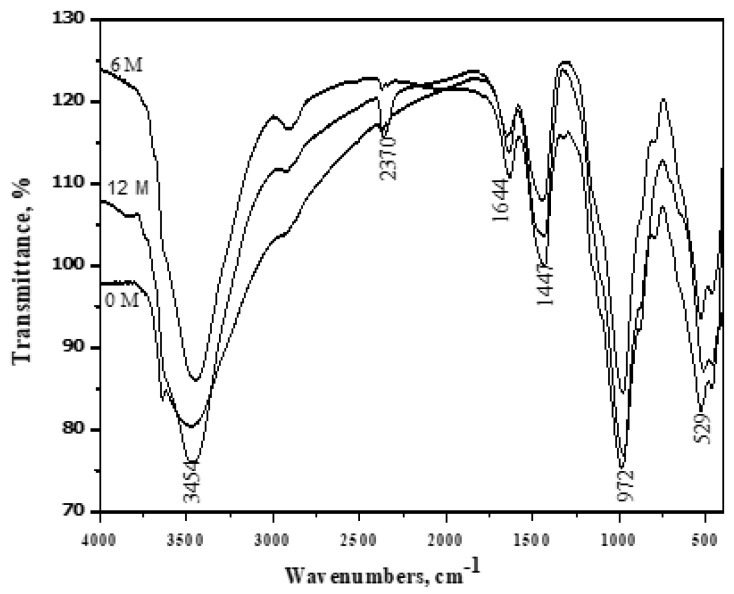
FT − IR spectroscopy of A4 (60% OPC + 40% GGBFS) immersed at 0, 6 and 12 months in seawater.

**Figure 12:**
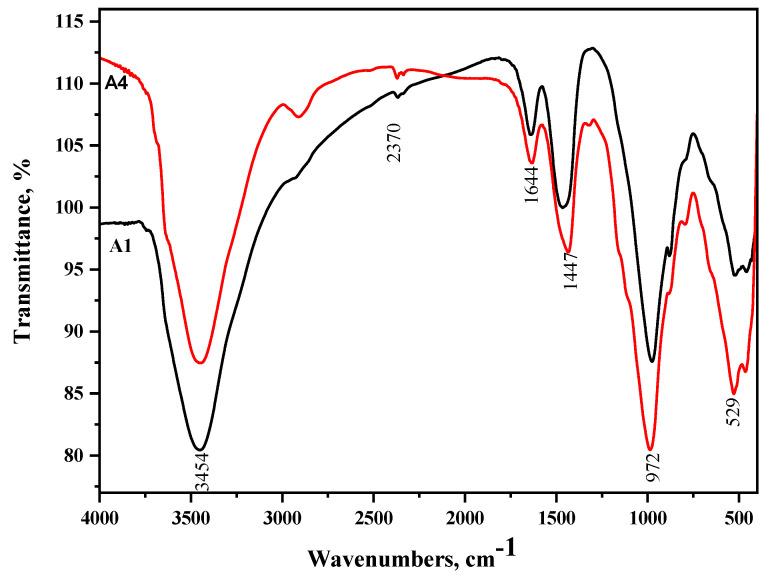
FT − IR spectroscopy of A1 and A4 cured at 6 months immersed in seawater.

**Figure 13:**
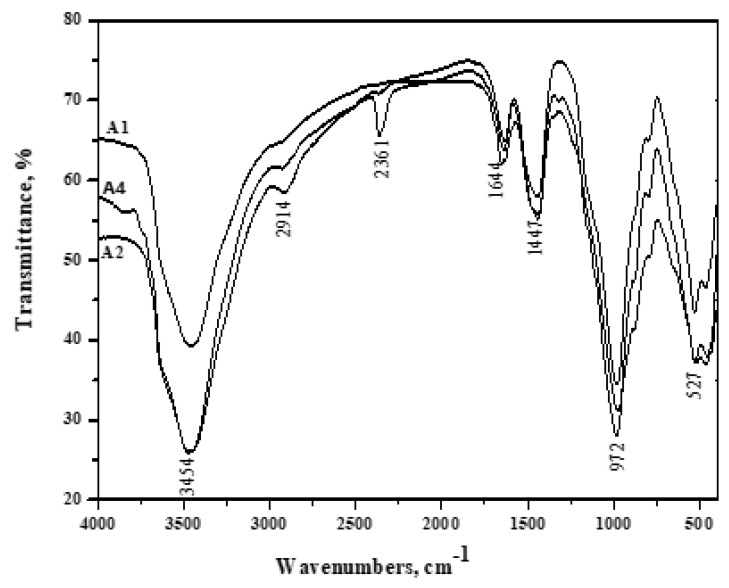
FT − IR spectroscopy of A1, A2 and A4 immersed for 12 months in seawater.

Figure 9, Figure 10, Figure 11, Figure 12 and Figure 13 display the FT-IR spectra of seawater-immersed hydrated slag cement pastes. Figure 9 exhibits the reduced intensity of the portlandite band at 3454 cm^−1^ after 12 months, resulting from GGBFS’s pozzolanic reaction with liberated portlandite during cement phase hydration. MgSO_4_ in seawater causes an acidic attack, converting Ca(OH)_2_ to gypsum and magnesium hydroxide. Moreover, MgCl_2_ undergoes a reaction with a fraction of Ca(OH)_2_, resulting in the formation of Mg(OH)_2_.

In Figure 10, the decreased intensity of the portlandite band at 3454 cm^−1^ in Mix A1 compared to Mix A4 could be attributed to two factors. Firstly, this is due to the dilution effect of pozzolana. Secondly, it results from the pozzolanic reaction of pozzolana phases with portlandite generated during cement phase hydration.

### 3.9. Thermal Analysis of Cement Pastes Immersed in Seawater

**Figure 14:**
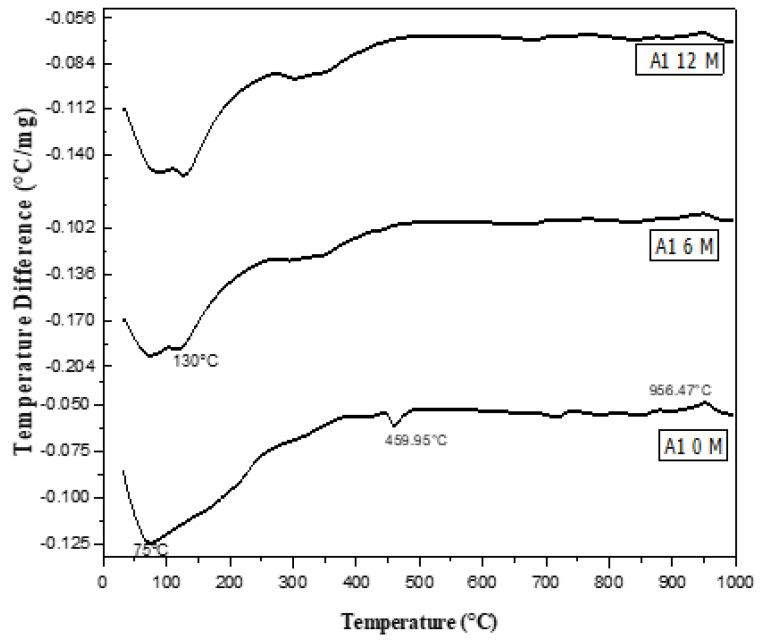
Differential Thermal Analysis (DTA) thermograms of A1 (30% OPC + 70% GGBFS) immersed in seawater for 0, 6 and 12 months.

**Figure 15:**
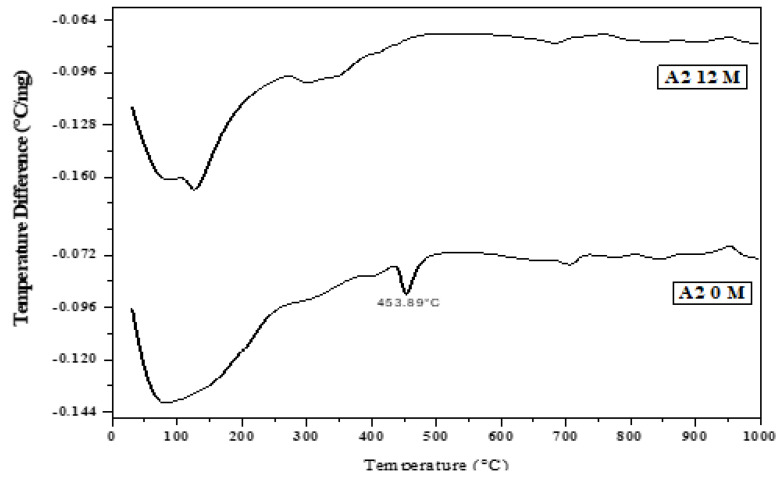
DTA thermograms of A2 (40% OPC + 60% GGBFS) immersed in seawater for 0 and 12 months.

**Figure 16:**
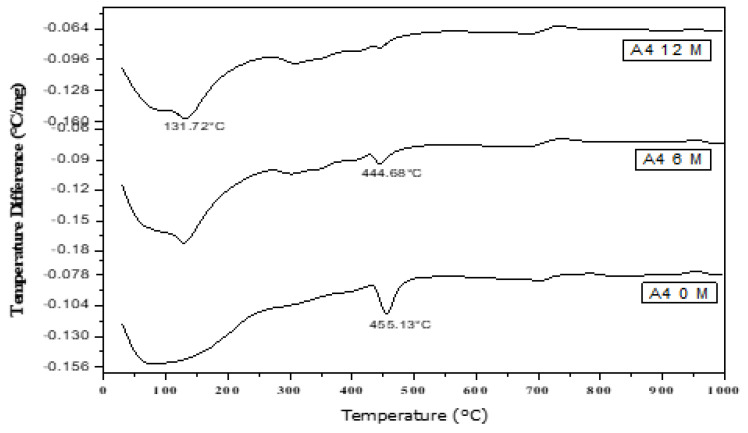
DTA thermograms of A4 (60% OPC + 40% GGBFS) immersed in seawater for 0, 6 and 12 months.

**Figure 17:**
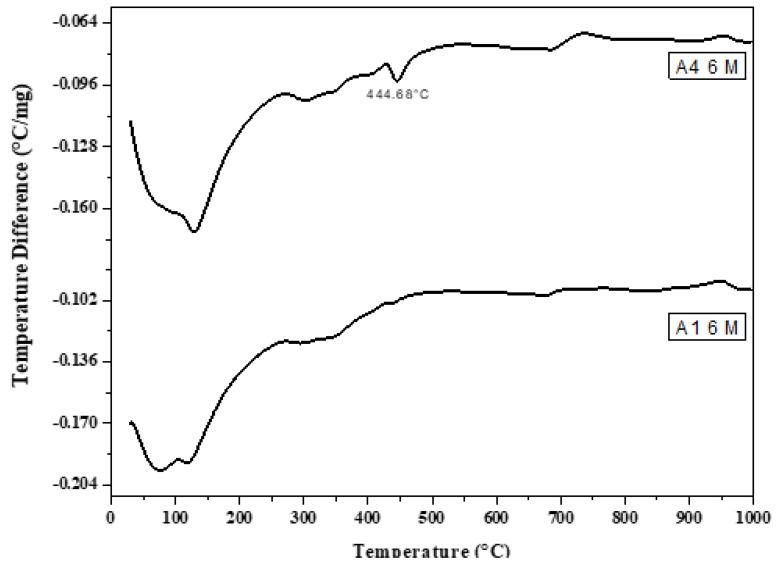
DTA thermograms of A1 and A4 cured at 6 months immersed in seawater.

**Figure 18:**
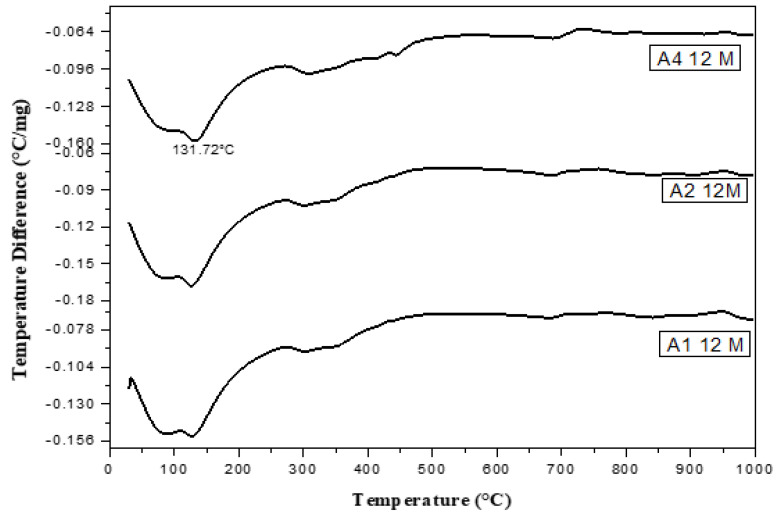
DTA thermograms of A1, A2 and A4 immersed for 12 months in seawater.

DTA (Figure 14, Figure 15, Figure 16, Figure 17 and Figure 18) can be employed to investigate the impact of seawater attack on the hydration products of GGBFS cement pastes when immersed in seawater for up to one year. The thermograms present various endothermic peaks and one exothermic peak at approximately 955 °C (Figure 14, Figure 15, Figure 16, Figure 17 and Figure 18). The lower endotherms for up to 300 °C are attributed to the decomposition of C-S-H gel, ettringite, and calcium chloroaluminate hydrate. There were three additional endothermic peaks at 316, 428, and 468 °C, which could be related to the decomposition of C2ASH_8_, Mg(OH)_2_ (brucite), and Ca(OH)_2_, respectively. Two endothermic peaks in the 700–900 °C range indicated amorphous and crystalline CaCO_3_ calcination. The exothermic peak at 955 °C is characteristic of C-S-H gel, which develops during the crystallization of monocalcium silicate CaO.SiO_2_ (wollastonite). At zero time, the thermogram of Mix A1 (Figure 14) showed the presence of C-S-H gel, AFt, C2ASH_8_, and hydrogarnet series at 315 °C. There is a characteristic endothermic peak of both Ca(OH)_2_ and CaCO_3_ and an exothermic peak for the crystallization of wollastonite [68,69].

A sample of A1 paste at six months in seawater showed the activation of seawater on the formation of hydration products. The amounts of chloroalumiate and sulfoaluminate hydrate with C-S-H gel and hydrogarnet series increased with a decrease in the endotherm of C-S-H gel as well as Ca(OH)_2_ due to the reaction of MgCl_2_ and NaCl_2_ with Ca(OH)_2_ to form Mg(OH)_2_, as small endotherm at 438 °C. As the immersion time increased, the amounts of hydration products and the hydrogarnet series and Mg(OH)_2_ increased with the disappearance of Ca(OH)_2_. This was also due to the continuous reaction of chloride with Ca(OH)_2_. Endothermic peaks due to carbonation were also detected. The exothermic peak at 955 °C was also present and sharp at zero time (in tap water). Then, the intensity and the sharpness of the exothermic peak decreased. This was also due to the attack of chloride or sulfate ions on the C-S-H gel, as shown in Equation (1):

3CaO·SiO_2_·3H_2_O + MgCl_2_ → Mg(OH)_2_ + CaCl_2_ + SiO_2_ gel (1)

Therefore, the amount of C-S-H gel decreased with the CaCl_2_ content due to increased chloroaluminate hydrate. Hence, the C-S-H gel was reduced at later ages (12 months), as seen in DTA thermograms.

Figure 18 shows the DTA thermograms of the immersed cement pastes A1, A2, and A4 in seawater for 12 months. The thermograms show the same hydrated products as the previous figures—endothermic peaks at lower temperatures up to 300 °C. The first peak at 100 °C was due to the decomposition of C-S-H gel, whereas that located up to 200 °C was mainly due to the chloroaluminate hydrate. The endotherm at ≈ 300–350 °C was primarily attributed to the presence of CAH and gehlenite hydrate C2ASH8. Also, A1, A2, and A4 contained 70, 60, and 40% (GGBFS), respectively. The significant difference between these three endothermic peaks was the amount of C-S-H gel formed from the activation of GBFS, i.e., as the amount of (GGBFS) increased, the exothermic peak increased accordingly. This also indicates that the exothermic peak is characteristic of the recrystallization of CaO.SiO_2_ (wollastonite). Conversely, the peak area of Mg(OH)_2_ at about 436 °C decreased with the slag content. Therefore, slag-rich cement is more durable or resistant to seawater attack.

**Figure 19:**
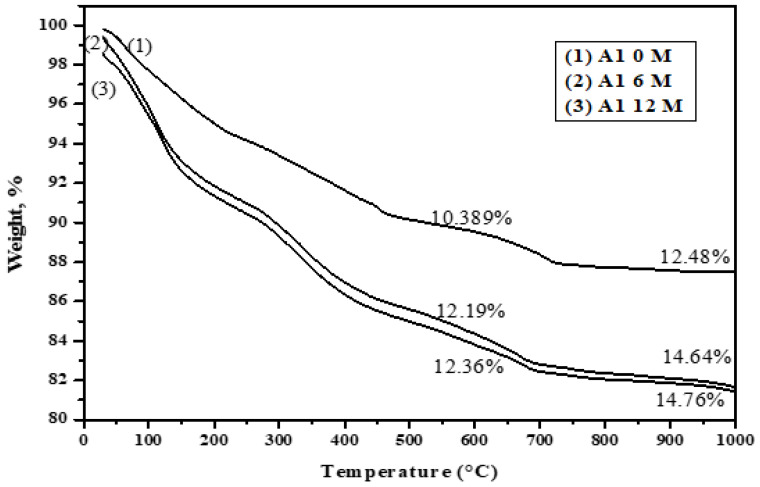
TGA thermograms of A1 (30% OPC + 70% GGBFS) immersed in seawater at 0, 6 and 12 months.

**Figure 20:**
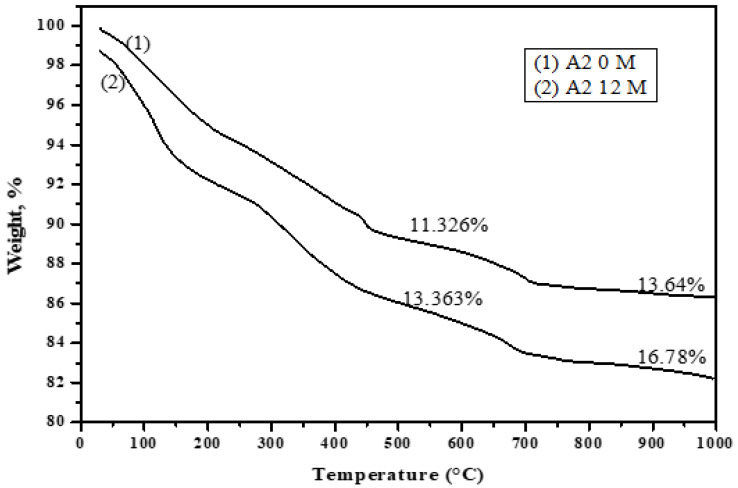
TGA thermograms of A2 (40% OPC + 60% GGBFS) immersed in seawater at 0 and 12 months.

**Figure 21:**
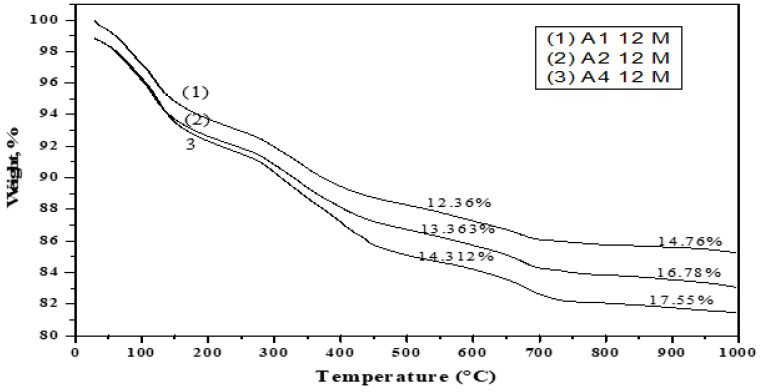
TGA thermograms of A1, A2 and A4 cured at 12 months under seawater.

**Figure 22:**
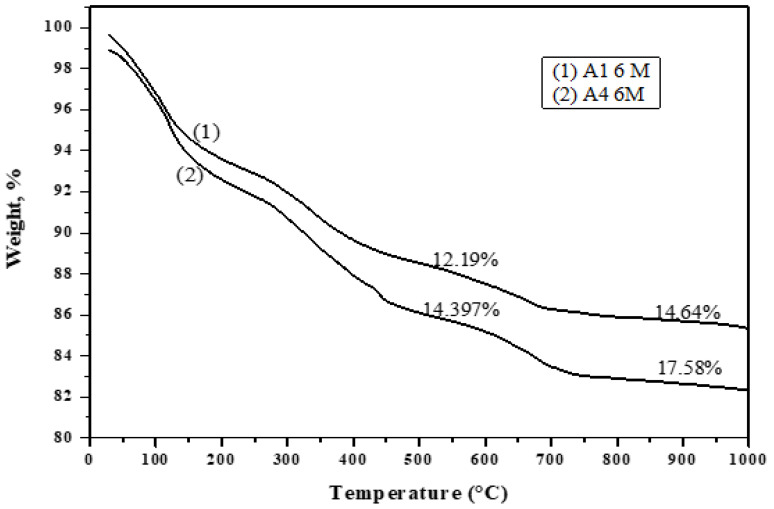
TGA thermograms of A1 and A4 cured at 6 months under seawater.

Figure 19, Figure 20, Figure 21 and Figure 22 depict the thermogravimetric (TG) profiles of hydrated GGBFS cement pastes when subjected to seawater curing for a duration of up to 12 months. A reduction in weight loss was evident with a higher slag content, which can be attributed to GGBFS exhibiting a lower hydraulic reactivity compared to OPC, resulting in enhanced cement paste durability.

### 3.10. X-ray Diffraction (XRD) Analysis

**Figure 23:**
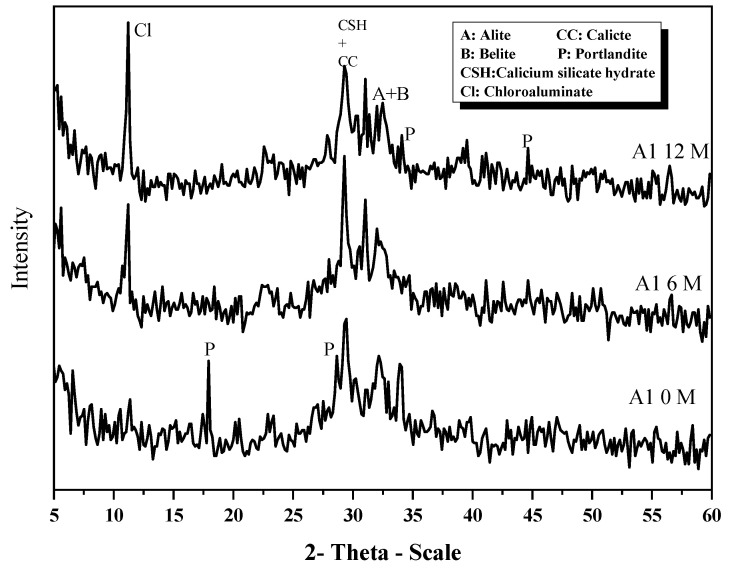
XRD patterns of A1 at 0, 6 and 12 months under seawater.

Figure 23 displays the (XRD) spectra of A1 at time intervals of 0, 6, and 12 months. The observed (XRD) patterns indicate that the presence of Ca(OH)_2_, CaCO_3_, C-S-H gel, β-C2S, and C3S diminished with an increasing immersion time due to the ongoing hydration process. It is noteworthy that calcium chloroaluminate hydrate did not form during immersion in tap water at the initial stage (zero time). However, with prolonged hydration, there was a noticeable increase in the intensity of the peak corresponding to calcium chloroaluminate hydrate. Additionally, the complete consumption of CH (calcium hydroxide) occurred after six months of immersion, which can ascribed to the pozzolanic interaction that occurred between GGBFS and portlandite. Throughout the 12-month period, anhydrous phases such as β-C2S and C3S remain present. Furthermore, the formation of C-S-H gel is observed, which overlaps with the calcium chloroaluminate hydrate peak.

## 4. Conclusions

1. The water content in all cement pastes demonstrated an incremental rise as the duration of immersion progressed.
2. These findings indicate a gradual decrease in the (FLC%) of GGBFS blends during up to one year of seawater exposure.
3. The BD and compressive strength of hardened blends demonstrate a progressive rise in value as both the curing time and slag content increased.
4. Drawing upon these findings, one can deduce that A2 (comprising 60% GGBFS and 40% OPC) represents the optimal pozzolanic cement, achieving a compressive strength of 85.7 MPa after one year of seawater immersion.
5. The outcomes derived from DTA, FT-IR, and XRD methodologies align well with each other, as well as with the observed chemical and physico-mechanical properties, indicating a strong consistency in these findings.
6. Regarding resistance against aggressive water attacks, it is evident that blended slag cement containing 60 wt. % GGBFS can effectively withstand durability threats.

## Figures and Tables

**Table 1:** Chemical analysis of the initial materials (wt. %).

Constituents		SiO_2_	Al_2_O_3_	Fe_2_O_3_	CaO	MgO	Na_2_O	K_2_O	SO_3_	LOI	Total
Initial materials	GGBFS	34.35	10.08	1.65	41.80	6.95	0.48	0.67	2.31	0.06	98.35
	OPC	20.51	5.07	4.39	62.21	2.00	0.23	0.29	2.25	2.40	99.35

**Table 2:** Mix proportions presented in weight percentages.

Blend ID	GGBFS, %	OPC, %	W/C, %
A1	70	30	25
A2	60	40	26.5
A4	40	60	28

## Data Availability

Not applicable.
